# Publisher Correction: An optimized workflow for single-cell transcriptomics and repertoire profiling of purified lymphocytes from clinical samples

**DOI:** 10.1038/s41598-020-62066-z

**Published:** 2020-04-14

**Authors:** Richa Hanamsagar, Timothy Reizis, Mathew Chamberlain, Robert Marcus, Frank O. Nestle, Emanuele de Rinaldis, Virginia Savova

**Affiliations:** 1 Sanofi Immunology and Inflammation Research Therapeutic Area, 270 Albany St, Cambridge, MA 02139 USA; 2 New York University College of Arts and Sciences, 32 Waverly Pl, New York, NY 10003 USA

Correction to: *Scientific Reports* 10.1038/s41598-020-58939-y, published online 10 February 2020

The original version of this Article contained an error in Affiliation 1, which was incorrectly given as ‘Sanofi Immunology and Inflammation Research Therapeutic Area, 270 Albany St, Cambridge, MA, 02139, England’.

The correct affiliation is listed below:

Sanofi Immunology and Inflammation Research Therapeutic Area, 270 Albany St, Cambridge, MA, 02139, USA.

This error has now been corrected in the HTML and PDF versions of the Article.

