# Supplementary material for: An optimized workflow for single-cell transcriptomics and repertoire profiling of purified lymphocytes from clinical samples
Source: Sci Rep. 2020 Feb 10;10:2219. doi: 10.1038/s41598-020-58939-y (PMC7010687; doi:10.1038/s41598-020-58939-y)
Supplement: Supplementary file 4 — Supplementary Information 4. [file 41598_2020_58939_MOESM4_ESM.pdf]

Title

An optimized workflow for single-cell transcriptomics and repertoire profiling of purified lymphocytes from clinical samples.

Authors:

Richa Hanamsagar[1], Timothy Reizis[2], Mathew Chamberlain[1], Robert Marcus[1], Frank O. Nestle[1], Emanuele de Rinaldis[1], and Virginia Savova[1]

Affiliations:

[1]Sanofi Immunology and Inflammation Research Therapeutic Area, 270 Albany St, Cambridge, MA 02139.

[2]New York University College of Arts and Sciences, 32 Waverly Pl, New York, NY 10003.

Corresponding Author:

Virginia Savova, Ph. D.

Sanofi

Principal Senior Scientist/Lab Head

Precision Immunology

Immunology & Inflammation Research Therapeutic Area

virginia.savova@sanofi.com

TEL.: 617.866-9313

Supplementary Figure 1

A

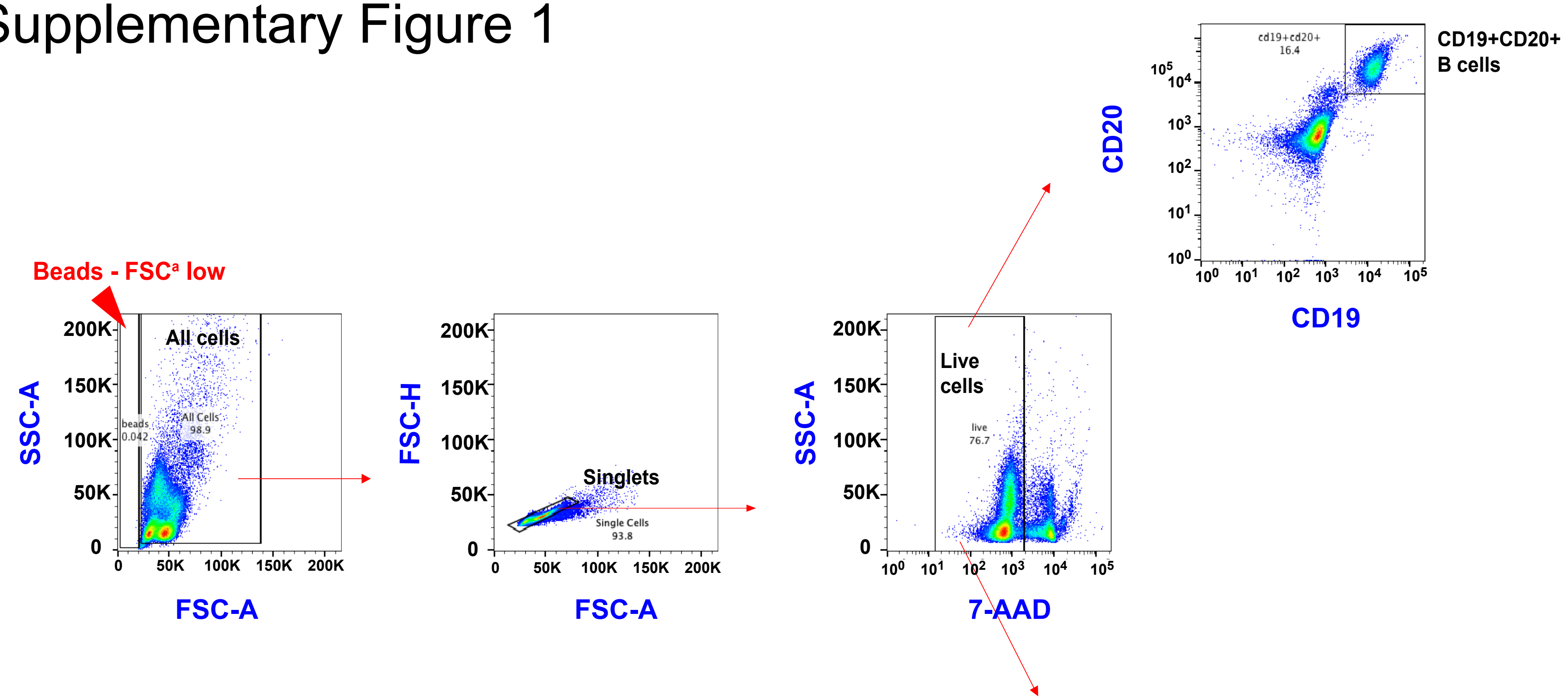

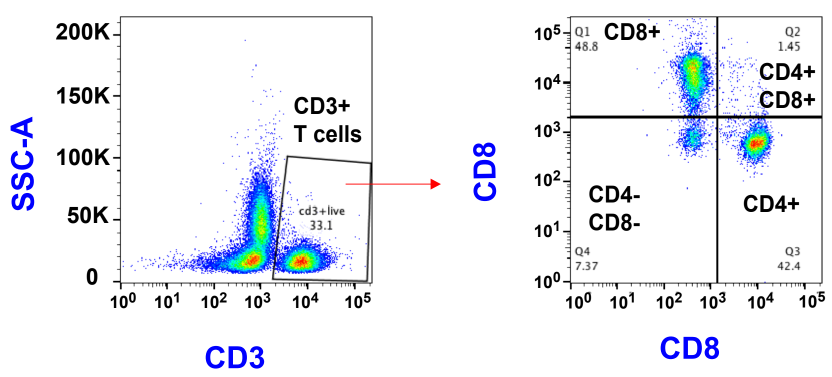

B

Panel								
**Antibody**	**Flourophore**	**ex**	**em**	**company**	**Cat#**	**Lot#**	**ul/test**	**x 10 tests**
CD19	APC	650nm	660nm	Biolegend	392504	B257010	5ul	50ul
CD20	PE-Cy7	496nm	785nm	Biolegend	302312	B257732	5ul	50ul
Stain Buffer				BD Biosciences	554656	7283909	10ul	100ul
**Total**							**20 ul**	**200 ul**
**Antibody**	**Flourophore**	**ex**	**em**	**company**	**Cat#**	**Lot#**	**ul/test**	**x 10 tests**
CD3	BUV737	348nm	737nm	BD Biosceince	564307	7335654	5ul	50ul
CD4	FITC	494nm	520nm	Biolegend	357406	B204828	5ul	50ul
CD8	BV-510	405nm	510nm	BD Biosciences	56319	818357	5ul	50ul
Stain Buffer				BD Biosciences	554656	7283909	15ul	150ul
**Total**							**30ul**	**300ul**

Supplementary Figure 2

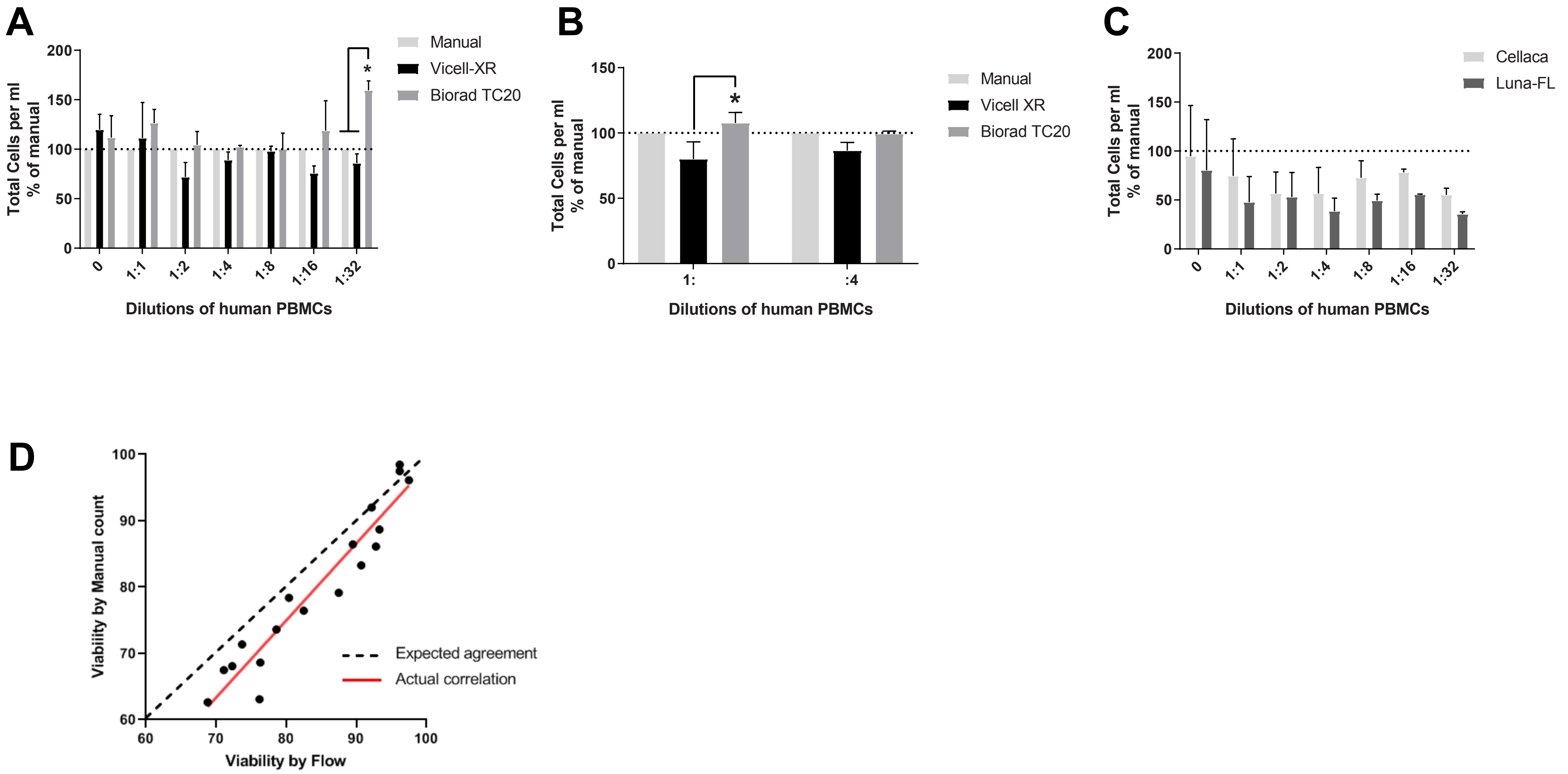

Supplementary Figure 3

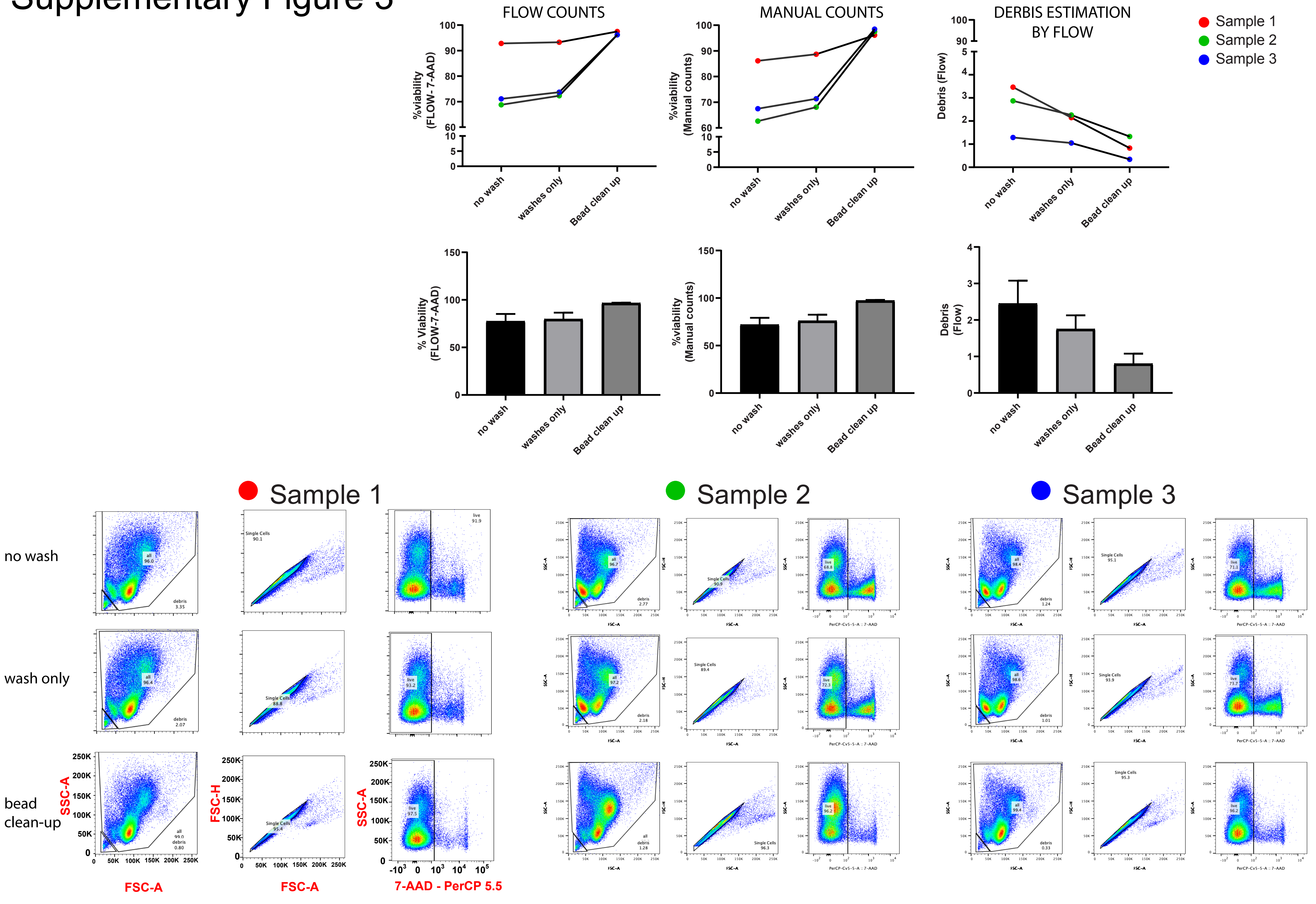

Supplementary Figure 4

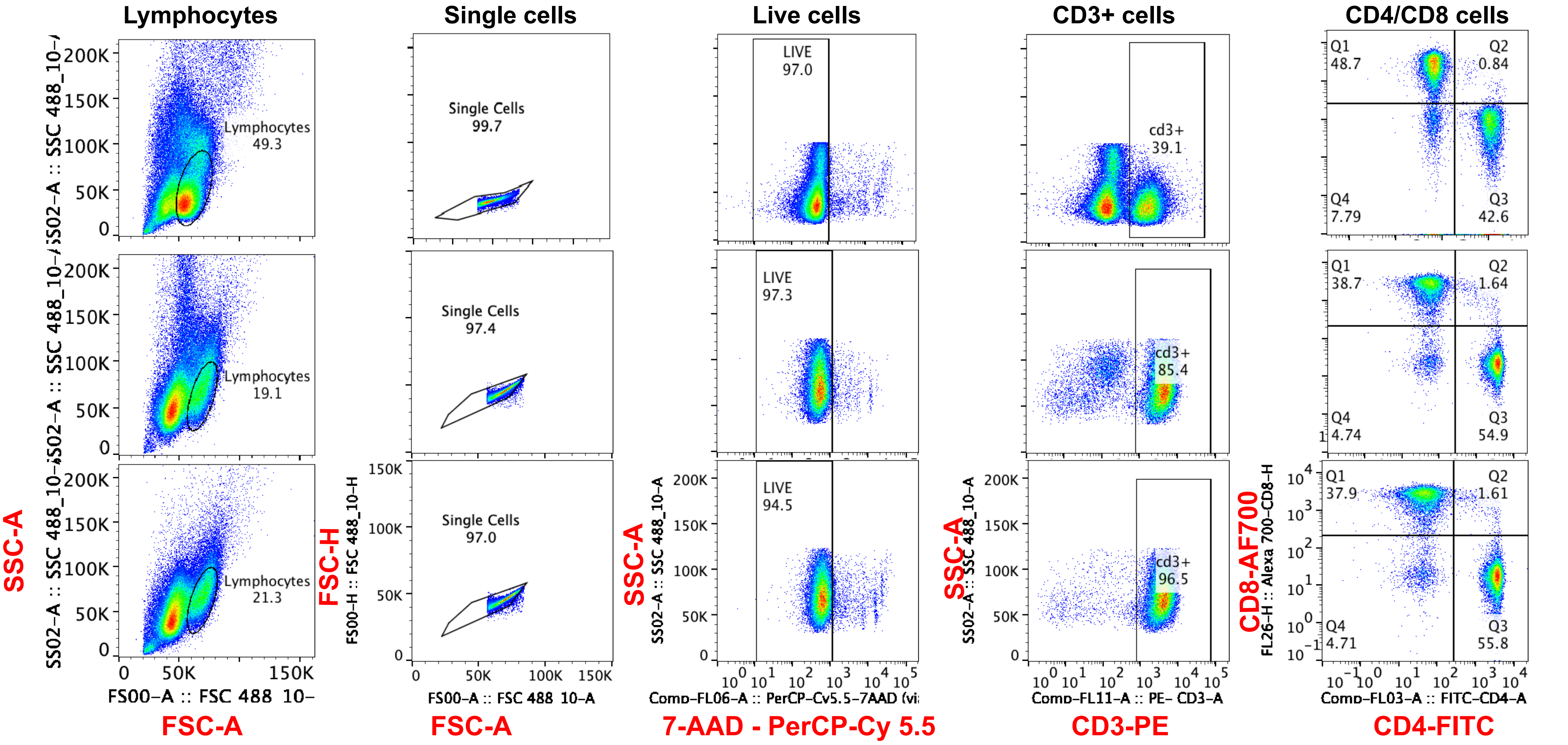

Alternate gating strategy for lymphocytes with no change in results:

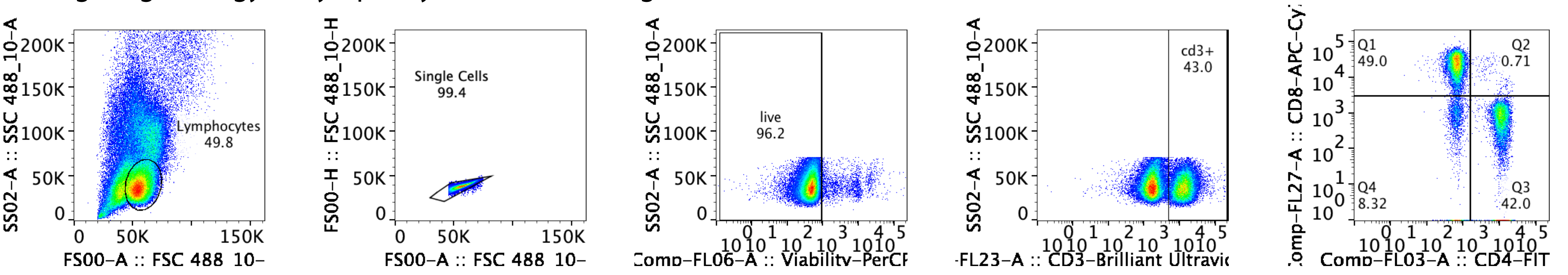

Supplementary Figure 5

A

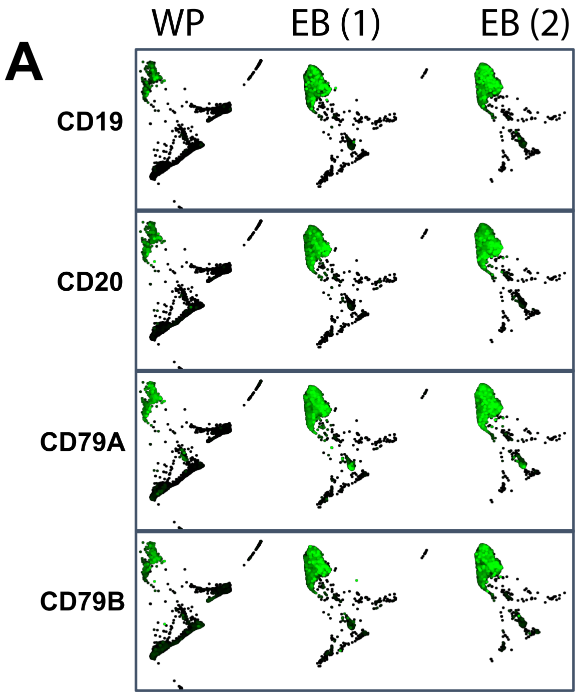

Human B cell markers gene expression

B

Clonotype	Naive B cells	Memory B cells	Plasma Cells	"Pre-B cells"	T cells
CL38	0	7	0	0	0
CL47	0	0	7	0	0
CL55	0	0	5	0	0
CL7	0	0	4	0	0
CL2	1	0	0	4	3
CL42	5	0	0	0	0
CL65	5	0	0	0	0
CL56	6	0	0	0	0
CL45	7	0	0	0	0
CL46	7	1	0	0	0

Number of cells expressing clonotype across different cell subtypes
